# CO_2_ efflux from subterranean nests of ant communities in a seasonal tropical forest, Thailand

**DOI:** 10.1002/ece3.1255

**Published:** 2014-09-24

**Authors:** Sasitorn Hasin, Mizue Ohashi, Akinori Yamada, Yoshiaki Hashimoto, Wattanachai Tasen, Tomonori Kume, Seiki Yamane

**Affiliations:** 1 Department of Forest Biology, Faculty of Forestry, Kasetsart UniversityBangkok, 10900, Thailand; 2 Center for Advanced Studies in Tropical Natural Resources, NRU-KU, Kasetsart UniversityBangkok, 10900, Thailand; 3 School of Human Science and Environment, University of HyogoHimeji, Hyogo, 670-0092, Japan; 4 Graduate School of Fisheries Science and Environmental Studies, Nagasaki UniversityNagasaki, 852-8521, Japan; 5 Division of Phylogenetics, Museum of Nature and Human Activities, University of HyogoSanda, 669-1546, Japan; 6 School of Forestry and Resource Conservation, National Taiwan UniversityTaipei, 106-17, Taiwan; 7 Department of Earth and Environmental Sciences, Faculty of Science, Kagoshima UniversityKagoshima, 890-0065, Japan

**Keywords:** ant nest, carbon dioxide, environmental factor, ground-dwelling ant, tropical forest

## Abstract

Many ant species construct subterranean nests. The presence of their nests may explain soil respiration “hot spots”, an important factor in the high CO_2_ efflux from tropical forests. However, no studies have directly measured CO_2_ efflux from ant nests. We established 61 experimental plots containing 13 subterranean ant species to evaluate the CO_2_ efflux from subterranean ant nests in a tropical seasonal forest, Thailand. We examined differences in nest CO_2_ efflux among ant species. We determined the effects of environmental factors on nest CO_2_ efflux and calculated an index of nest structure. The mean CO_2_ efflux from nests was significantly higher than those from the surrounding soil in the wet and dry seasons. The CO_2_ efflux was species-specific, showing significant differences among the 13 ant species. The soil moisture content significantly affected nest CO_2_ efflux, but there was no clear relationship between nest CO_2_ efflux and nest soil temperature. The diameter of the nest entrance hole affected CO_2_ efflux. However, there was no significant difference in CO_2_ efflux rates between single-hole and multiple-hole nests. Our results suggest that in a tropical forest ecosystem the increase in CO_2_ efflux from subterranean ant nests is caused by species-specific activity of ants, the nest soil environment, and nest structure.

## Introduction

Soil respiration is the total CO_2_ efflux from the soil surface, a major component of the carbon cycle in an ecosystem (Luo and Zhou 2006). Accurate estimations of total soil CO_2_ efflux are important to calculate the net carbon exchange between the atmosphere and terrestrial ecosystems (Raich and Potter 1995). Because tropical forests play a more significant role than other ecosystems in global carbon dynamics (Raich and Schlesinger 1992), it is important to understand the mechanisms and factors controlling total soil CO_2_ efflux in tropical forests. The total soil CO_2_ efflux fluctuates with changes in temperature and moisture (e.g., Xu and Qi 2001). In temperate and boreal ecosystems, temperature affects total soil CO_2_ efflux more strongly than does moisture, whereas in tropical ecosystems, the opposite situation can occur (Malhi et al. 1999). In particular, in tropical seasonal forests, total soil CO_2_ efflux clearly fluctuates with changes in soil moisture because there are distinct dry and wet seasons characterized by large differences in rainfall (Kume et al. 2012). The total soil CO_2_ efflux includes the CO_2_ produced by all living soil organisms; plant roots, soil microbes, and animals (Luo and Zhou 2006). Hanson et al. (2000) reviewed respiration sources to elucidate the mechanisms of total soil CO_2_ efflux. However, this review only covered the contribution of roots and the rhizosphere, and most of the data were obtained from temperate and boreal forests. Thus, data on the sources, or the classes of sources, of CO_2_ contributing to total soil CO_2_ efflux in the tropics are relatively limited, with few studies investigating the contribution of soil animals (Ohashi et al. 2008).

In some ecosystems, some ant taxa are important ground-dwelling arthropods in terms of species richness and biomass (Hölldobler and Wilson 1990; Folgarait 1998). Ants have been defined as ecosystem engineers, because they affect ecosystem processes and soil function by their nesting and foraging activities (Lavelle and Spain 2003). In their nests, ants alter their physical and chemical environments by constructing tunnels and chambers, accumulating soil particles, and storing litter and food above and below the tunnels in the ground nests (Folgarait 1998). Studies have reported that mound-type nests made from soil and/or litter have nutrient profiles that differ from those of surrounding soil or forest floor (Ohashi et al. 2007a; Domisch et al. 2008). Moreover, specific fauna and flora are found at ant mounds (Berg-Binder and Suarez 2012; Laakso and Setälä 1997). Similarly, subterranean ant nests can affect water infiltration rates (Lavelle and Spain 2003) and soil erosion (Cerdà and Jurgensen 2008) by decreasing soil bulk density and increasing soil porosity. The alteration of soil chemical properties, such as nutrient concentrations (Wagner et al. 2004), can result in different vegetation types and biodiversity in areas around subterranean nests (Wagner et al. 1997; Whitford et al. 2008). Some recent studies have suggested that the high CO_2_ efflux from ant mounds increases the spatial variability in total CO_2_ efflux from the forest floor (Domisch et al. 2006; Ohashi et al. 2005). This would make it more difficult to estimate the stand-scale average of total soil CO_2_ efflux. Ohashi et al. (2012) reported that the contribution of the mound CO_2_ efflux increased with forest stand age and suggested that ants affect variations in the C balance in ecosystems.

Recent studies reported that in the large litter-mound nests of *Formica* spp. CO_2_ effluxes were up to 12 times higher compared with surrounding soils in boreal forests (Domisch et al. 2006; Ohashi et al. 2005, 2007b), a subalpine forest (Risch et al. 2005), and in a marsh area (Wu et al. 2013). Similarly, CO_2_ efflux rates are up to five times higher in the soil-mound nests of *Solenopsis invicta* in pasture soil (Bender and Wood 2003), in nest mounds of *Lasius flavus* and *L. niger* in wetlands (Wu et al. 2013), and nests of *Acromyrmex balzani* in coastal plains (Sousa-Souto et al. 2012). These studies show that ant nests increase the CO_2_ efflux from soil, but most of them have been conducted in northern forests and grasslands where a few ant species build mound-type nests.

In tropical forests, ant biomass constitutes >10% of the total animal biomass (Wilson 1990), suggesting ants may play an important role in soil carbon dynamics. Furthermore, in Asian tropical forests, >2000 ant species have been recorded (Folgarait 1998). In these tropical forest ecosystems, it is possible that the CO_2_ efflux from ant nests is highly variable compared with those of other ecosystems. Based on the findings of “hot spots of total soil CO_2_ efflux” in a Bornean tropical forest, Ohashi et al. (2007c, 2008) proposed that these hot spots had been caused by CO_2_ emission from hidden subterranean ant nests under the measuring points. To our knowledge, no study has directly investigated CO_2_ efflux from ant nests in a tropical forest, probably because of difficulties in systematically measuring CO_2_ efflux from subterranean nests.

Ants could affect nest CO_2_ efflux in many ways such as directly via respiration and indirectly by altering soil properties and the environment, changing the respiration rate of other CO_2_ sources (Sousa-Souto et al. 2012; Ohashi et al. 2007b). In *Formica* ant mounds, the higher temperatures and lower moisture content compared with the surrounding soil enhanced the activity of ants and other organisms resulting in larger CO_2_ effluxes (Ohashi et al. 2007b). Likewise in tropical forests, the subterranean ant nests may increase CO_2_ efflux, not only by their respiration, but also by the changes in soil condition from nest construction, but no study clarified the factors that correlate to nest CO_2_ efflux.

We aimed to elucidate the CO_2_ efflux from subterranean nests in a tropical seasonal forest ant community, Thailand. In particular, we focused on: (1) comparing CO_2_ efflux of the nest and the surrounding soil; (2) the variation of nest CO_2_ efflux among ant species; (3) the relationship between the soil environment of the ant nests and nest CO_2_ effluxes; and (4) the potential impacts of nest structure on the CO_2_ efflux from subterranean nests.

## Materials and Methods

### Study sites

The study was conducted in a dry evergreen forest (DEF) at Sakaerat Environmental Research Station (SERS; 14°30′N, 101°56′ E, 500 m a.s.l.) in northeastern Thailand. The DEF covers 64% of the natural forest area at the SERS (Trisurat 2009). The study area had a gentle slope of less than 10°. The forest site consisted of *Hopea ferrea* and *H. odorata*, forming a closed canopy with heights ranging from 23 to 40 m. The mid-layer comprised *Hydnocarpus ilicifolius*, *Aglaia pirifera*, *Walsura trichostenon,* and *Memecylon caeruleum*, which formed a canopy at a height of 16–22 m. The lower canopy, which was 4–14 m in height, consisted of *M. ovatum*, *Ixora barbata*, and *Randia wittii* (Lamotte et al. 1998). The forest floor was covered with a thin layer of undergrowth containing seedlings from the three different forest canopy species. The mean litter mass accumulated on the forest floor (A0-layer) was 25 t ha^−1^ (dry weight) and included leaves, twigs, and dead wood (Yamada et al. 2003). The thickness of litter layer was 2–5 cm. The soil texture was loam and clay loam, derived from sandstone (Lamotte et al. 1998), classified as ultisols soil (USDA classification). The soil porosity and available water capacity in the 0 to 50-cm depth layer were in the range of 50–67% and 6–24 mm, respectively (Murata et al. 2009). The mean annual precipitation, temperature, and relative humidity at the SERS meteorological station were 978 mm, 26.3°C, and 88.3%, respectively, from 2000 and 2009. The climate is characterized by a dry season from November to May, (<50 mm rainfall per month) and a wet season from June to October (Sakurai et al. 1998). We conducted our research from October, 2010 to September, 2011. During the measurement period, the mean monthly precipitation, temperature, and relative humidity were 43.5 mm, 25.5°C, and 76%, respectively, in the dry season (November–May) and 159.7 mm, 27.1°C, and 82.3%, respectively, in the wet season (June–October). The annual precipitation, temperature, and relative humidity during this period were 1237 mm, 25.8°C, and 81.7%, respectively.

### Subterranean ant nests

For our CO_2_ efflux measurements, we choose 13 dominant ant species with high abundances and activity based on preliminary observations. Among the 13 ant species, the ant worker body length ranged from 1.5 to 17 mm (Table1). We identified the entrance holes of potential nests using the food-baiting method. We did not select nests close to large trees, rotten logs, or stones, to decrease variations in CO_2_ efflux due to CO_2_ production from other sources. We ensured that the sampled ant nests were constructed from soil, to ensure that the soil characteristics at the nest were comparable to those of surrounding soil. Ant workers were collected from the entrance holes of each nest for identification. Ants were identified to the subfamily and genus level according to Bolton (1994) and to the species level by comparisons with the ant collections in the Ant Museum at Kasetsart University (AMK), Thailand. After identifying each species, we selected the main entrance hole of each nest by observing worker traffic intensity. To identify nest-hole type (single-hole or multiple-hole types), we searched for other entrance holes around the main entrance hole, collecting ants from the adjacent and main entrance holes, and allowing them to fight each other in a chamber to determine whether they were from the same nest (Heller et al. 2006). We selected three to six independent nests for each of the 13 species, making a total of 61 nests (Table1). We established an experimental plot 2 × 2 m in the area of each nest. The plot centered the main entrance hole and included all of the other entrance holes. We measured the diameter of each entrance and then calculated the average entrance hole diameter for each nest. The nest structure of subterranean ants is characterized by the size and number of tunnels and chambers (Tschinkel 2003). Therefore, it is possible that the size and number of entrance holes are related to the nest structure characteristics. In this study, the mean diameter and number of the nest entrance holes per plot were used to calculate a nest structure index.

**Table 1:** Ant species, number, and characteristics of the ant nests examined in this study. The mean ant body size and hole diameter are shown with the standard error in parentheses. Range is shown for the number of entrance holes

Species	Species Code	Number of ant nests^1^	Ant body size^2^ (mm)	Number of nest holes	Hole diameter^3^ (mm)
*Anochetus graeffei* Mayr, 1870	A1	4 [1,3]	4.27 (0.03)	1	4.1 (0.3)
*Anochetus* sp.2 of AMK	A2	4 [1,3]	4.93 (0.02)	1	4.5 (0.2)
*Anoplolepis gracilipes* (F. Smith, 1857)	AG	5 [2,3]	4.83 (0.03)	1–3	37.3 (1.0)
*Aphaenogaster* sp.1 of AMK	AP	6 [3,3]	5.41 (0.04)	1	9.9 (0.4)
*Diacamma* cf. *vagans* (F. Smith, 1860)	DV	5 [2,3]	9.82 (0.11)	1	12.8 (0.8)
*Ectomomyrmex astuta* (F. Smith, 1858)	EA	4 [1,3]	16.34 (0.28)	1–2	4.9 (0.1)
*Harpegnathos venator* (F. Smith, 1858)	HV	3 [3,0]	12.80 (0.11)	1	18.3 (0.9)
*Odontoponera denticulata* (F. Smith, 1858)	OD	6 [3,3]	9.51 (0.08)	1–3	3.6 (0.2)
*Odontomachus rixosus* F. Smith, 1857	OR	6 [3,3]	10.85 (0.05)	1-2	43.5 (6.4)
*Pheidole hongkongensis* Wheeler, 1928	PH	4 [1,3]	2.50 (0.00)	1–2	1.6 (0.1)
*Pheidole plagiaria* F. Smith, 1860	PP	5 [2,3]	3.49 (0.01)	1	48.8 (1.6)
*Pheidole parva* Mayr, 1865	PV	4 [1,3]	1.70 (0.03)	1	1.3 (0.2)
*Tetramorium lanuginosum* Mayr, 1870	TL	5 [2,3]	2.47 (0.03)	1	1.9 (0.1)
Total		61			

1 The number in square brackets means number of nests in wet and dry season.

2 15–30 individuals were randomly collected from the nests, and the length from head to last section of abdomen was measured using microscope.

3 The number of replicates was the number of ant nests.

### Measurements of CO_2_ efflux and environmental factors

Nest CO_2_ efflux was measured from the soil surface at the entrance hole of the nests, using a commercial respiration chamber (SRC-1, PP-system; Amesbury, MA) and infrared gas analyzer (EGM-4, PP-systems) following methods in Ohashi et al. (2007c, 2008). Additionally, we selected five to six soil control points surrounding the nest entrance holes in each experimental plot for the soil CO_2_ efflux measurement. The average distance between nest holes and control locations was 36±41 SD cm. We examined the presence/absence of ant nests and/or other ants and termites by digging up the soil under the control points to a depth of 10–30 cm, following the completion of all measurements to confirm there was no influence from the ant nest. The absence of nest was used as the criterion to define where there is no impact of ant nest.

CO_2_ efflux measurement has performed using a closed-chamber method. We inserted PVC collars (height 3–4 cm, diameter 10 cm) into the soil at least 0.5-cm deep to mount the commercial respiration chamber and put plasticine sealing between the collars and soils to make them airtight during the measurement. The collars were set up 1 day before the CO_2_ measurement and left in the place throughout the experiment. To minimize ant activity disturbance, CO_2_ measurements were started at the main entrance hole. We then measured the other entrance holes and controls in the same plot and averaged the nest and soil CO_2_ efflux, respectively. Measurements were repeated three times at each entrance hole and twice for the controls and then averaged for each measurement point. It took around 15 min for a nest and 10 min for a control point to finish the repetition and obtain a data of CO_2_ efflux.

After CO_2_ measurements, soil temperature and moisture content were measured at three locations around each collar. We measured the soil temperature at a depth of 10 cm with a Drip-Proof Type Digital Thermometer (MODEL PC-9215; SATO, Tokyo, Japan) and from ground level to 6 cm with a moisture sensor (ThetaProbe type ML2x; Delta-T Devices Ltd., Cambridge, UK).

The series of measurements were conducted during the day, between 09:00 and 16:00 h, with measurements at each plot taking approximately 2 h, resulting in two to three plots measured daily. We measured at least three nests randomly for each species. The measurements of most ant species occurred during the wet and dry seasons, except for *Harpegnathos venator* (Table1).

### Statistical analyses

The differences in CO_2_ effluxes among ant nests and the control soils, season and ant species were examined using a general linear model (GLM) analysis with the sampling location (ant hole and surrounding soil) as within-subject factor, and season (wet and dry) and ant species as between-subject factors. Raw data were natural log-transformed to decrease heteroscedasticity, after checking for normality and homogeneity using Shapiro–Wilk's and Levene's tests, respectively. Whenever significant results (at the level of *P* < 0.05) occurred in the GLM, a post hoc test was performed using Bonferroni pairwise comparisons. To compare the relationship between CO_2_ efflux and environmental factors (i.e., soil temperature and soil moisture content), we used linear regression analyses for the ant nest and the control data separately.

We used the number of entrances and the diameter of entrance holes as an index of nest structure. The number of entrance holes was classified into two groups, single-(only one) and multiple (greater than one) hole types. The effect of the different hole type on the CO_2_ efflux from ant nests was determined using a two-way ANOVA with the hole types and season as explanatory variables. Nest CO_2_ efflux data were natural log-transformed to meet the assumptions of normality. The relationship between the mean diameter of entrance holes per nest and nest CO_2_ efflux was tested using linear regression analysis. All statistical analyses were performed with SPSS ver. 20.0.0 for Windows (SPSS Inc., Chicago, IL).

## Results

### CO_2_ efflux from ant nests in the wet and dry seasons

**Figure 1:**
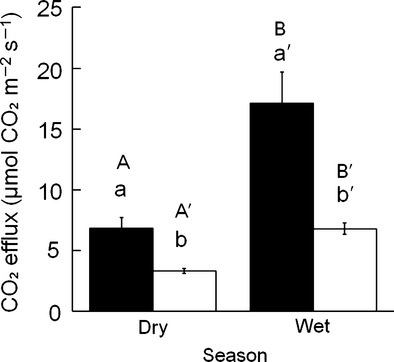
CO_2_ efflux in ant nests (black bars) and the surrounding soil (white bars) in the dry and wet season. Error bars represent standard error (n ≥ 25). Different lower case letters indicate significant differences between nests and soil for dry (a and b) and wet seasons (a′ and b′) (*P* < 0.001). Different capital letter indicates a significant difference between dry and wet seasons for ant nests (A and B) and for the surrounding soil (A′ and B′) (*P* < 0.001).

We measured CO_2_ efflux from 61 subterranean ant nests: 34 nests in the wet season and 27 nests in the dry season (Table1). In both seasons, the season-specific mean CO_2_ efflux rates from ant nests were significantly higher than those from the controls (Table2). The mean CO_2_ efflux rates from ant nests were 2.5 and 2.0 times higher than those of the controls in the wet and dry seasons, respectively (*P* < 0.001, Fig.1). There was a significant seasonal variation in CO_2_ efflux rates from the ant nests and the surrounding controls (Table2). The location-specific mean CO_2_ efflux rates were 2.6 and 2.1 times higher in the wet season than the dry season, in the nests and controls, respectively (*P* < 0.001, Fig.1). CO_2_ efflux rates from ant nests during the wet season ranged from 6.1 to 63.2 *μ*mol CO_2_ m^−2^ s^−1^, a larger range than that in the dry season (0.8–24.7 *μ*mol CO_2_ m^−2^ s^−1^). Similarly, CO_2_ efflux rates from the controls showed larger fluctuations during the wet season, 3.6–14.5 *μ*mol CO_2_ m^−2^ s^−1^, than during the dry season, 1.3–6.1 *μ*mol CO_2_ m^−2^ s^−1^, but the range of fluctuation was smaller than that of the nests.

**Table 2:** Comparison of CO_2_ efflux between location, season, and ant species

	CO_2_ efflux (*μ*mol CO_2_ m^−2^ s^−1^)			
Source of variation	*d.f.n*.	*d.f.d*.	*F*	*P*
Location	1	37	227.65	**0.001**
Seasons	1	37	63.65	**0.001**
Species	12	37	5.96	**0.001**
Location × Season	1	37	9.21	**0.001**
Location × Species	12	37	9.58	**0.001**
Species × Season	11	37	1.26	0.29
Location × Species × Season	11	37	1.10	0.39

Statistically significant *P*-values are in bold.

### Interspecies variations of CO_2_ efflux

**Figure 2:**
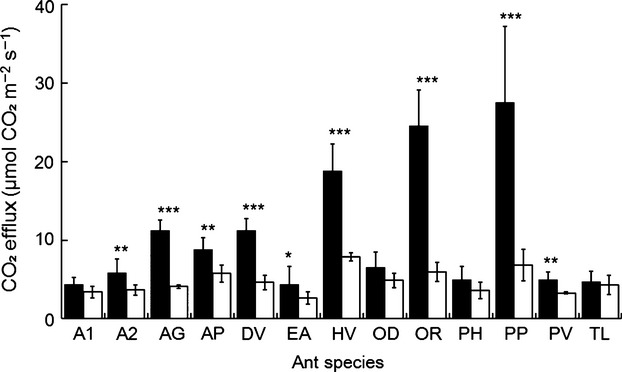
CO_2_ efflux in ant nests (black bars) and surrounding soil (white bars) for each species. Error bars represent standard error (*n* > 3). *, **, and ***(*P* < 0.05, *P* < 0.01, and *P* < 0.001, respectively) indicate significant differences between the nest and soil CO_2_ effluxes within the ant species.

Regardless of seasonality, species-specific mean CO_2_ efflux rates from the ant nests were significantly higher compared with the controls (*P* < 0.001, Table2). The mean CO_2_ efflux from ant nests varied from 4.3 (±0.9 SE, *n* = 4) in *A. graeffei* to 27.5 (±9.7 SE, *n* = 5) *μ*mol CO_2_ m^−2^ s^−1^ in *P. plagiaria*. The CO_2_ efflux from the controls was relatively stable, from 2.7 (±0.8 SE, *n* = 4) in *E. astuta* to 7.7 (±0.6 SE, *n* = 3) *μ*mol CO_2_ m^−2^ s^−1^ in *H. venator* (Fig.2). Pairwise comparisons showed significantly higher CO_2_ efflux rates from nests compared with the controls in nine of 13 ant species. These species were: *Anochetus* sp.2 of AMK, *A. gracilipes*, *Aphaenogaster* sp.1 of AMK, *D*. cf. *vagans*, *H. venator*, *O. rixosus*, *E. astuta*, *P. plagiaria*, and *P. parva* (Fig.2).

There was significantly greater CO_2_ efflux from *H. venator* nests than from nests of *O. denticulata*, *E. astuta, A. graeffei, P. hongkongensis*, and *T. lanuginosum* (Table3). CO_2_ efflux for *O. rixosus* and *P. plagiaria* was significantly higher than for most of the other species. Conversely, nest CO_2_ efflux for *A. graeffei*, *Anochetus* sp.2 of AMK, *Aphaenogaster* sp.1 of AMK, *O. denticulata*, *E. astuta*, *P. hongkongensis,* and *P. parva* was significantly lower than that of the other two or three species. Significantly lower CO_2_ efflux occurred in *T. lanuginosum* compared with *A. gracilipes*, *D*. cf. *vagans*, *H. venator*, *O. rixosus*, and *P. plagiaria*.

**Table 3:** Results of pairwise comparisons for CO_2_ efflux among ant species for ant nests (upper-right) and surrounding soil points (lower-left). Significant differences are given as *P*-values. NS represents not statistically significant results. Ant species abbreviations are presented in Table^1^

Ant species	A1	A2	AG	AP	DV	EA	HV	OD	OR	PH	PP	PV	TL
A1		NS	NS	NS	NS	NS	**0.02**	NS	**0.001**	NS	**0.001**	NS	NS
A2	NS		NS	NS	NS	NS	NS	NS	**0.01**	NS	**0.01**	NS	NS
AG	NS	NS		NS	NS	NS	NS	NS	NS	NS	NS	NS	**0.05**
AP	NS	NS	NS		NS	NS	NS	NS	**0.02**	NS	**0.01**	NS	NS
DV	NS	NS	NS	NS		NS	NS	NS	NS	NS	NS	NS	**0.04**
EA	NS	NS	NS	NS	NS		**0.01**	NS	**0.001**	NS	**0.001**	NS	NS
HV	NS	NS	NS	NS	NS	NS		**0.01**	NS	**0.05**	NS	NS	**0.001**
OD	NS	NS	NS	NS	NS	NS	NS		**0.001**	NS	**0.001**	NS	NS
OR	NS	NS	NS	NS	NS	NS	NS	NS		**0.001**	NS	**0.001**	**0.001**
PH	NS	NS	NS	NS	NS	NS	NS	NS	NS		**0.001**	NS	NS
PP	NS	NS	NS	NS	NS	NS	NS	NS	NS	NS		**0.001**	**0.001**
PV	NS	NS	NS	NS	NS	NS	NS	NS	NS	NS	NS		NS
TL	NS	NS	NS	NS	NS	NS	NS	NS	NS	NS	NS	NS	

Statistically significant *P*- values are in bold.

### Relationship between CO_2_ efflux, soil temperature, and soil moisture content

**Figure 3:**
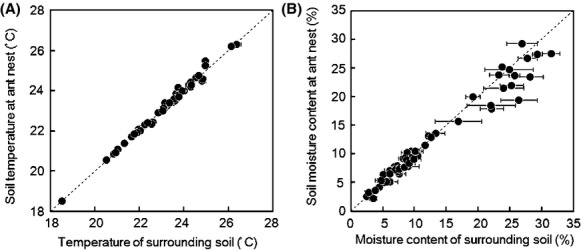
Comparison of the soil environment between the ant nest and their surrounding soil: temperature (A) and moisture content (B).

**Figure 4:**
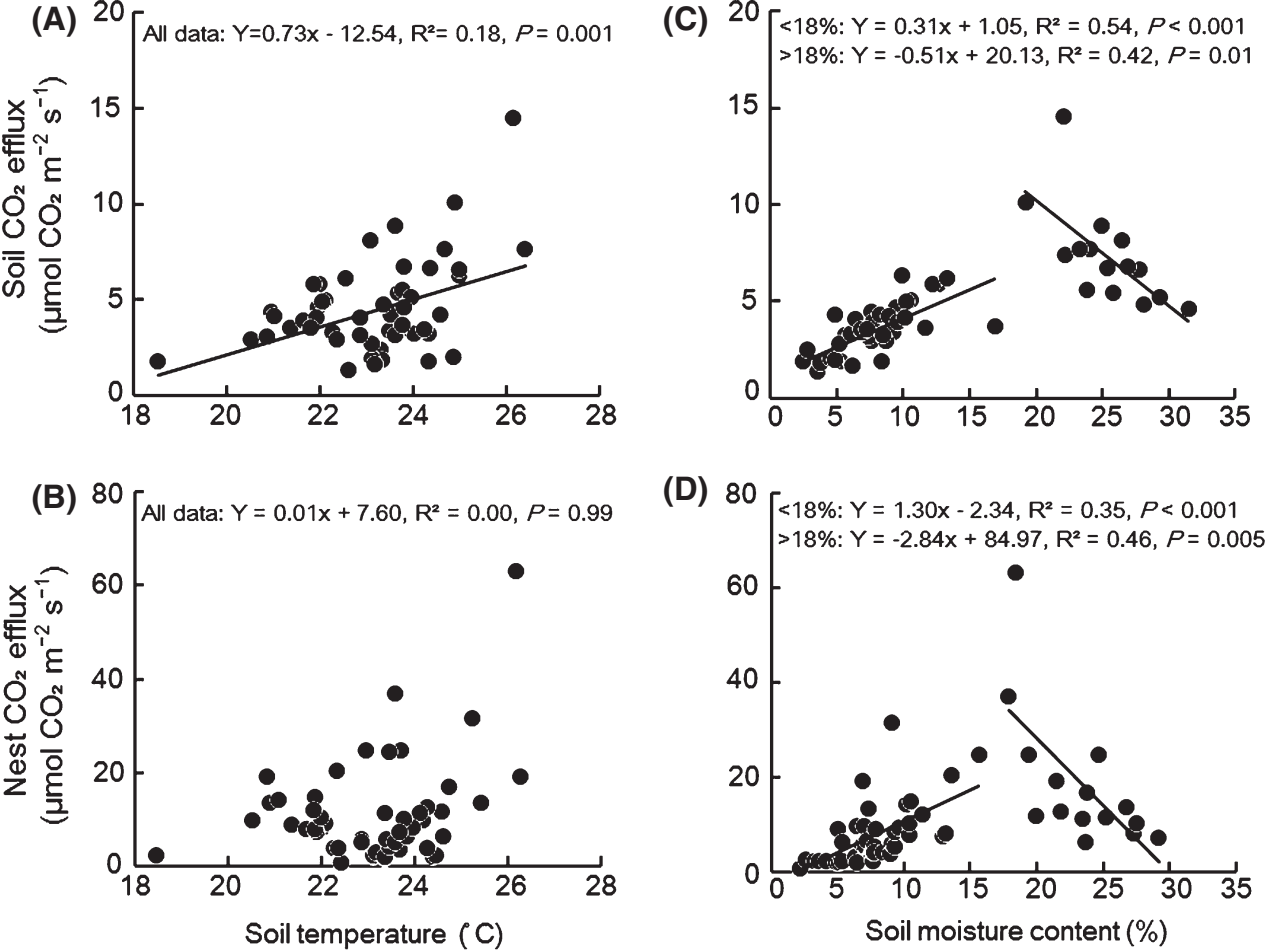
Changes in nest and soil CO_2_ efflux with the soil temperature in the nest (A) and surrounding soil (B), the soil moisture in the nest (C) and surrounding soil (D). The regression analysis for soil moisture content was run separately for soil moisture content was greater and less than 18%.

The large variations in soil temperature and moisture content were mainly related to the season (Fig.3A, B). Soil temperature and moisture content were similar for each of the ant nests and control pairs except those above 18%, where the soil moisture content was lower in the nests (Fig.3B). Seasonal changes in soil temperature and moisture affected the soil CO_2_ efflux. The linear regression analysis showed a significant positive relationship between CO_2_ efflux rates and temperature in the control (Fig.4A), but there was no significant relationship for ant nests (Fig.4B).

The relationship between moisture content and CO_2_ efflux rates changed around 18% soil moisture content. The regression analysis showed a significant positive relationship between CO_2_ efflux from ant nests and soil moisture content <18%, with a significantly negative relationship when the moisture content was >18% (Fig.4D). Similar results were obtained for the relationship between CO_2_ efflux and soil moisture content in the controls (Fig.4C). Interestingly, the regression coefficients were higher for the nests compared with the controls (Fig.4C, D).

### Impacts of the hole type and diameter on nest CO_2_ efflux

**Figure 5:**
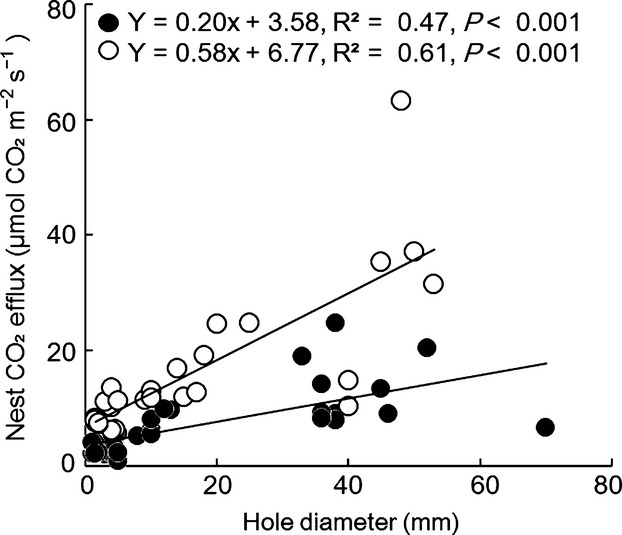
Relationship between nest entrance diameter and CO_2_ efflux. Black circles represent the dry season and white circles the wet season.

There was no significant difference in CO_2_ efflux rates between single-and multiple-hole type nests (hole type, *F*_1,58_ = 0.5, *P* = 0.48; season, *F*_1,58_ = 24.8, *P* < 0.001; interaction, *F*_1,58_ = 0.77, *P* = 0.39). There were significant positive relationships between CO_2_ efflux rates and hole diameter in the wet and dry season (Fig.5). The regression coefficient in the wet season was larger compared with that of the dry season.

## Discussion

Our results showed that the CO_2_ efflux from ant nests was significantly higher than that from the surrounding soil in a seasonal tropical forest (Fig.2). Similar effects of ant nests on soil CO_2_ efflux have been reported in boreal forests (Domisch et al. 2006; Ohashi et al. 2005, 2007b), subalpine forest (Risch et al. 2005), wetland (Wu et al. 2013), pasture (Bender and Wood 2003), and coastal plains (Sousa-Souto et al. 2012). Given that ecosystem structure is more complicated and biodiversity is greater in tropical forests than in other ecosystems (Allaby 2006), we expected larger variations in CO_2_ efflux from ant nests in tropical forests. Our results showed that there was significantly greater CO_2_ efflux from ant nests than from the surrounding soil, and that this CO_2_ efflux is ant species specific in a tropical forest.

We measured the CO_2_ efflux from subterranean-type ant nests, whereas other studies have focused on mound-type nests (e.g., Wu et al. 2013; Sousa-Souto et al. 2012; Ohashi et al. 2005). Even though mound-type nests are easy to find and are relatively common in their ecosystems, non-mound-type nests (i.e., subterranean nests) are more common in other ecosystems such as tropical forests (Hölldobler and Wilson 1990). To our knowledge, there are no reports on CO_2_ efflux from nonmound-type nests, probably because subterranean nests are difficult to find. There are many differences between subterranean and mound-type nests, including the nest structure, material of construction, relationships with other animals within the nest/mound, foraging behavior of the ants, and nest size (Hölldobler and Wilson 1990). These differences may result in different patterns, limitation factors, and different mechanisms of CO_2_ efflux between the two nest types. We found that moisture content and nest entrance diameter significantly affected nest CO_2_ efflux. Previous studies reported that moisture content did not affect CO_2_ efflux from mound-type nests, in contrast to the findings of this study (Wu et al. 2013; Ohashi et al. 2007b).

We found that CO_2_ efflux from nests was significantly higher than that from the surrounding control soil in the wet and dry seasons, but the difference was larger in the wet season than in the dry season. Variations in the magnitude of differences in CO_2_ efflux between nest and controls have been reported in boreal and subalpine forests, where ant mound CO_2_ efflux was 2–12 times higher than that from surrounding soils during the active ant time, but there was no difference in dormant ant times (Risch et al. 2005; Ohashi et al. 2007b; Domisch et al. 2006). These findings suggest that the nesting and forging activity of ants is an important factor in increasing CO_2_ efflux from nests. Because ants in tropical rain forests do not have a dormant period (Gove et al. 2005), it is plausible that these ants are active throughout the year in a warm climate (Allaby 2006). In our study ecosystem, the climate is warm enough for ants to remain active in both the wet and dry seasons, so the CO_2_ efflux differs between nests and the control throughout the year. However, ants may vary their activity between seasons, causing a seasonal change in CO_2_ efflux between nests and controls. For example, the size of the ant population may change between seasons, affecting the amount of ant-originated CO_2_. In the tropical forest, leaf litter containing food resources for the ants resulted in higher ant diversity/abundance during the wet season than the dry season (Kaspari and Weiser 2000; Hahn and Wheeler 2002). The increased food sources in the wet season would allow ants to establish new nests and the ant queen to produce more workers and increase in the production of reproductive caste, thus increasing the ant population (Hölldobler and Wilson 1990; Kaspari 2000). The larger ant population may increase nesting and foraging activity (Wagner et al. 2004), raising their metabolic activity (Rosengren et al. 1987), resulting in higher CO_2_ efflux. However there is no study about the impact of changes in ant population size on nest CO_2_ efflux. Future study is necessary to confirm the idea.

Our observations showed that soil moisture content was lower in the nest compared with their surrounding soil when the soil moisture range was 18–31%, mainly during the wet season (Fig.3B). The decrease in soil moisture could be explained by the soil modification from ant nesting activity (Lavelle and Spain 2003). Nest construction decreases soil bulk density and increases the number of soil macrospores with the size of tunnels and chamber within ant territories (Cerdà and Jurgensen 2008; Lobry de Bruyn 1999), allowing rapid water infiltration in ant nests compared with soils without nests (James et al. 2008; Cerdà and Jurgensen 2008; Whitford et al. 2008). At our study site, the soil consisted of dense loam/clay loam containing numerous micropores and small macropores, making water flow very slowly through this substrate. Therefore, the increase in macropores and the continuous porosity from ant nesting activity may have increased soil water drainage, decreasing soil moisture content at nest sites during the wet season.

Our results showed positive relationships between soil CO_2_ efflux and temperature (Fig.4A), similar to previous studies (e.g., Ohashi et al. 2008), but there was no significant relationship between soil temperature and nest CO_2_ efflux (Fig.4B). The different temperature effects between nest and soil could result from the differences in CO_2_ producers between ant nests and soil. The main sources of CO_2_ efflux from soil are soil microbes and plant roots (Schwendenmann et al. 2003; Adachi et al. 2006; Ohashi et al. 2008). Soil temperature is an important factor for microbes and roots activity, with studies on soil respiration reporting exponential and/or linear increases in soil CO_2_ efflux with increasing temperature (Luo and Zhou 2006). In many tropical systems, soil temperature is not a strong predictor of soil CO_2_. In this study, R^2^ value for the relationship between soil CO_2_ efflux and temperature was only 0.18 (Fig.4A). Given that tropical seasonal forests in this region have constantly high temperatures with little variation compared with other climate regions (Hashimoto et al. 2007), the slight changes in temperature may not have a significant impact on ant activity. Therefore, no clear relationship occurred between CO_2_ efflux from ant nests and temperature. We found positive and negative relationships between CO_2_ efflux and soil moisture content both in the ant nests and the surrounding soil (Fig.4C, D), even though the source of CO_2_ production may differ between the nest and soil. The effects of soil moisture content, both negative and positive, under relatively high and low moisture conditions, respectively, in tropical forests have been reported (Schwendenmann et al. 2003; van Straaten et al. 2009). These results suggest that there may be the most preferable moisture content for the CO_2_ producers in soil in these ecosystems and if soil moisture content increase or decrease more than the most preferable content, the amount of CO_2_ production starts to decrease, as we found in this study. Our results suggest that all of ant, soil microbe, and root activity may be controlled by soil moisture content. However, the steeper regression slope for the nest CO_2_ efflux (Fig.4D) suggests that ants are more sensitive to soil moisture content compared with the source of CO_2_ efflux from surrounding soils.

**Figure 6:**
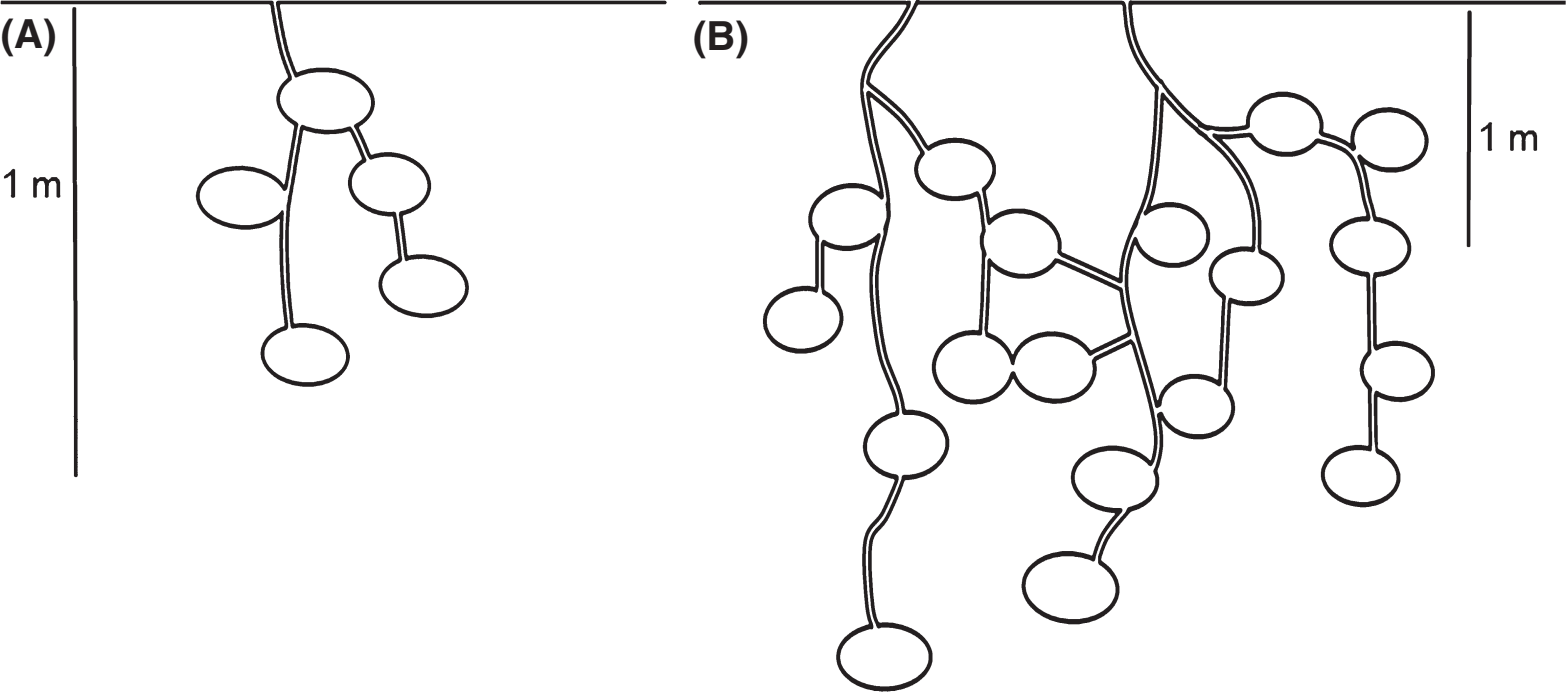
Examples of the structure of subterranean ant nests. Simple nests consisted of a single large chamber and a small horizontal chamber connected with a vertical and horizontal tunnel (A). Complex nests consisted of multiple small chambers with long narrow tunnels connecting each chamber (B). These illustrations were modified from Tschinkel (2003) based on observations from our study.

CO_2_ efflux from ant nests was highly variable among the different ant species (Fig.2). Significantly higher CO_2_ efflux occurred in three ant species, *H. venator*, *O. rixosus,* and *P. plagiaria* than those of other 5–8 species*,* significantly while lower efflux was recorded in eight other species, *A. graeffei*, *Anochetus* sp.2 of AMK, *Aphaenogaster* sp.1 of AMK, *O. denticulata*, *E. astuta*, *P. parva*, *P. hongkongensis*, and *T. lanuginosum* than those of other 2–5 species (see Fig.2, Table3). Nest structure may explain the difference, as the structure of subterranean nests varies depending on the ant species (Tschinkel 2003). After our experiments, we excavated all of the nests to see the nest structure (data not shown). We found that three ant species *H. venator*, *O. rixosus*, and *P. plagiaria*, with higher nest CO_2_ efflux than others, built a simple nest with a big chamber and straight tunnels of relatively short distances (2 to 10 cm) (Fig.6A). In contrast, the eight species that emit less nest CO_2_ flux built a complex nest with many small chambers and long narrow tunnels connecting the chambers (Fig.6B). The straight tunnel of the three former species would facilitate CO_2_ efflux, but CO_2_ probably fail to emerge from the narrow complex tunnels and chambers in the eight (latter) species resulting in higher within nest concentrations.

The relationship between entrance hole diameter and nest CO_2_ efflux from ant nests (Fig.5) supported the idea that nest structure is an important factor in nest CO_2_ efflux variations. There may be other reasons for these variations, including the number of ants in the colony, ant body size and behavior, indirect effect of ants on other CO_2_ sources, and the phenology of each colony.

To understand the mechanisms of nest CO_2_ efflux more clearly, future research should focus on the colony characteristics (e.g., population size and behavior), the respiration rates of different-sized ants, and the relationship between ant species and other CO_2_ sources. Furthermore, we need to understand the impact of nest occupation area on the larger scale for each ant species and its variation to estimate the impact of ant nests on total soil CO_2_ efflux at the ecosystem level under current and future climate conditions. Our result creates a paradigm for future studies of the mechanisms of total soil CO_2_ efflux in tropical forests.
